# Supplementary material for: Effects of tuberculosis and/or HIV-1 infection on COVID-19 presentation and immune response in Africa
Source: Nat Commun. 2023 Jan 12;14:188. doi: 10.1038/s41467-022-35689-1 (PMC9836341; doi:10.1038/s41467-022-35689-1)
Supplement: Supplementary file 1 — Supplementary Information [file 41467_2022_35689_MOESM1_ESM.pdf]

Comorbid tuberculosis and/or HIV-1 infection and COVID-19 presentation and immune response in Africa: Supplementary data

1. Supplementary Table 1: Final diagnoses in non-COVID-19 control participants.
2. Supplementary Table 2: COVID-19 outcome in relation to presenting features.
3. Supplementary Table 3: Clinical features of COVID-19 participants with tuberculosis and HIV-tuberculosis.
4. Supplementary Table 4: Characteristics of HIV-1 uninfected and infected persons with either immune evidence of tuberculosis sensitization but no symptoms (latent tuberculosis, LTBI) or microbiologically-confirmed pulmonary tuberculosis recruited to previous studies.
5. References.
6. Supplementary Figure 1: Type, number, and frequency and types of co-morbidity present in COVID-19 and ODC participants.
7. Supplementary Figure 2: Relationships between radiographic scores.
8. Supplementary Figure 3: Radiographic appearances of combined SARS-CoV2 and tuberculosis infection in the presence or absence of HIV-1 co-infection.
9. Supplementary Figure 4: Percentage of peripheral lymphocytes CD4 and CD8 positive in relation to COVID-19 severity and the presence or absence of HIV-1 and/or tuberculosis co-morbidities.
10. Supplementary Figure 5: CD8 T cell response to SARS-CoV-2 antigens

Supplementary Table 1

	Disease	n	HIV-1 co-infected
Non communicable disease (n=20)	Chronic cardiac failure	7	0/7
	Exacerbation asthma / COPD	6	1/6
	Diabetic keto-acidosis	3	0/3
	Cerebrovascular accident	1	0/1
	Gastro-intestinal	2	1/2
	Pulmonary embolism	1	0/1
Infectious diseases (n=13)	HIV-1	13	-
	Tuberculosis	5	5/5
	Pneumocystis jirovecii pneumonia	5	5/5
	Community acquired pneumonia	2	0/2
	Urinary tract infection	1	0/1
Unclear final diagnosis at discharge*		2	0/2
Outpatients from COVID testing centre (n=7)	Lower respiratory tract infection symptoms	7	1/7

Supplementary Table 1: Final diagnosis of non-COVID-19 participants.

*COVID-19 actively excluded

Supplementary Table 2

	**Survived** **71.2%** (n=74)	**Died** **28.8%** (n=30)
**Age** (median, IQR)	52 [44-57]	55 [46-66]
**Male** (%, n)	54% (n=40)	76.7% (n=23)
**HIV-1 co-infected** (%, n)	33.8% (n=25)	20% (n=6)
on antiretroviral therapy (ART)	72% (n=18)	83.3% (n=5)
Time on ART (years) [a]	9.5 [6-12]	10 [3.5-11]
CD4 count (cells/mm^3) [a]	144 [53-332]	113 [45-270]
Log Viral load [a]	<1.3 [<1.3-4]	3.17 [<1.3-5.21]
***M. tuberculosis* positive** (%, n)	12% (n=9)	20% (n=6)
Previous tuberculosis episode/s (within 5 years)	22.2% (n=2)	33.3% (n=2)
**Co-morbidities**		
Cardiovascular	6.7% (n=5)	6.7% (n=2)
Hypertension	44.6% (n=33)	56.7% (n=17)
Diabetes	36.5% (n=27)	46.7% (n=14)
Obesity	29.3% (n=22)	33.3% (n=10)
Other respiratory diseases	9.2% (n=7)	-
**SARS-CoV-2 serology positive**[b]	67.6% (n=50)	73.3% (n=22)
Cut-off index (median, IQR)	5.5 [0.29-21.95]	13.4 [0.43-35.6]
**WHO COVID-19 ordinal scale at enrolment (%, n)**		
3	22.6% (n=18)	-
4	44% (n=33)	17.2% (n=5)
5	22.6% (n=17)	44.8% (n=13)
6	8.1% (n=6)	36.7% (n=11)
7	-	3.4% (n=1)
**Severe (WHO ≥5)**	31.1% (n=23)	83.3% (n=25)
**Cycle threshold SARS PCR** (n=85) [a]	31.5 [26.8-34]	29.1 [25.4-33.6]
**On steroid treatment (%, n)**	73% (n=54)	93.3% (n=28)
**Overall days in clinical care** [a]	11 [6-23]	15 [7-22]

Supplementary Table 2: COVID-19 Outcome in relation to presenting features.
IQR: interquartile range
[a]: Median and [IQR]
[b]: SARS-CoV-2 serology was performed using the Roche Elecsys assay, measuring SARS-CoV-2 nucleocapsid-specific antibodies. Results are reported as numeric values in form of a cut-off index (signal sample/cut-off), where a COI < 1.0 corresponds to non-reactive plasma and COI ≥ 1.0 to reactive plasma.

Supplementary Table 3

Patient number	Age	HIV-1 status	CD4 /mm^3	Viral Load	on ART Y/N	Method of TB diagnosis	Time between + SARS CoV-2 PCR and TB dx	Prior TB episodes	Time since last TB episode	WHO score at enrolment	Outcome
9	37	-ve	NA	NA	NA	sputum Xpert + (Rif S)	simultaneous	2	3 years	4	discharged
36	43	-ve	NA	NA	NA	sputum Xpert + (Rif S)	TB dx 25 days after	0	NA	5	discharged
57	37	-ve	NA	NA	NA	sputum culture + (after 24 days; sputum Xpert ND)	TB dx 6 weeks prior	0	NA	3	discharged
58	43	-ve	NA	NA	NA	sputum Xpert + (Rif S)	simultaneous	0	NA	4	discharged
73	41	-ve	NA	NA	NA	sputum Xpert + (Rif R)	simultaneous	0	NA	6	died
80	61	-ve	NA	NA	NA	sputum Xpert + (Rif S)	TB dx 20 days after	0	NA	7	died
125	65	-ve	NA	NA	NA	sputum Xpert + (Rif S)	simultaneous	0	NA	6	died
3	31	+ve	106	ND	N	pleural fluid Xpert + (Rif S)	simultaneous	0	NA	3	discharged
6	34	+ve	110	17870	Y	sputum Xpert + (Rif S)	TB dx 3 months prior	0	NA	4	discharged
61	37	+ve	26	523463	Y	clinical diagnosis: disseminated TB	TB dx 18 days prior	1	2 years	4	died
93	43	+ve	51	2941	Y	sputum Xpert + (Rif S), sputum auramine 3+	simultaneous	1	4 months	4	died
95	49	+ve	106	15860	N	sputum Xpert + (Rif S) and culture positive (Rif S)	simultaneous	0	NA	3	discharged
97	55	+ve	17	201574	N	pericardial fluid and sputum Xpert<br>sputum Xpert + (Rif S); urine LAM +	simultaneous	0	NA	3	discharged
104	56	+ve	209	LDL	Y	sputum Xpert + (Rif S)	TB dx 21 days after	0	NA	5	died
152	27	+ve	ND	395	Y	urine LAM positive, urine Mtb culture positive (TTP 20 days, Rif R, INH S)	simultaneous	2	1 year	3	discharged

Supplementary Table 3: Clinical features of COVID-19 participants with tuberculosis and HIV-tuberculosis.
Diagnoses of COVID-19 and TB occurring within 5 days of each other were denoted “simultaneous”.
+ve: positive, -ve: negative, dx: diagnosis, INH: isoniazid, LAM: Lipoarabinomannan, LDL: Lower than Detectable Limit, Mtb: *Mycobacterium tuberculosis*, NA: not applicable, ND: not done, Rif: Rifampicin, R: resistant, S: sensitive, TTP: time to positivity

	**HC (HIV-/aTB-)**	**HIV+/aTB-**	**HIV-/aTB+**	**HIV+/aTB+**
n	72	29	28	34
Age [a]	32 [26-38]	34 [32-42]	33 [28-47]	37 [32-45]
Male (%)	48.6%	24.1%	71.4%	73.5%
CD4 count (cells/mm^3) [a]	nd	481 [358-700]	nd	236 [121 - 355]
Log HIV viral load[a]	na	<1.3 [<1.3-4.18]	na	4.49 [2.19-5.00]
On ART (%)	na	80.6 %	na	38.9%
Unaffected Lung (%) [a]	na	na	50 [30-70]	60 [30-90]
CRP (μg mL^{-1}) [a]	1 [1-4]	3 [2-10]	100 [27.5-115]	72 [36-123]

Supplementary Table 4: Characteristics of healthy controls (HC) and mono- and co-infected HIV-1 (HIV+) and microbiologically confirmed pulmonary tuberculosis (TB+) controls, recruited to previous studies [1,2].

ART: Anti-retroviral treatment, CRP: C-reactive protein, nd: not done, na: not applicable.

[a] Medians and [interquartile range]

Supplementary References

1. Du Bruyn, E., *et al. Mycobacterium tuberculosis*-specific CD4 T cells expressing CD153 inversely associate with bacterial load and disease severity in human tuberculosis. *Mucosal Immunol* **14**, 491-499 (2021).
2. Riou, C., *et al.* Disease extent and anti-tubercular treatment response correlates with *Mycobacterium tuberculosis*-specific CD4 T-cell phenotype regardless of HIV-1 status. *Clin Transl Immunology* **9**, e1176 (2020).

Supplementary Figure 1

Co-morbidities

Hypertension (n=50/23)
Diabetes (n=41/12)
Obesity (n=34/14)
HIV-1 (n=31/13)
Active tuberculosis (n=15/5)
Cardiovascular (n=7/18)
Other respiratory (n=7/11)
Kidney disease (n=4/0)
Malignancy (n=3/0)
Immunosuppressive therapy (n=1/1)
Transplant recipient (n=1/0)

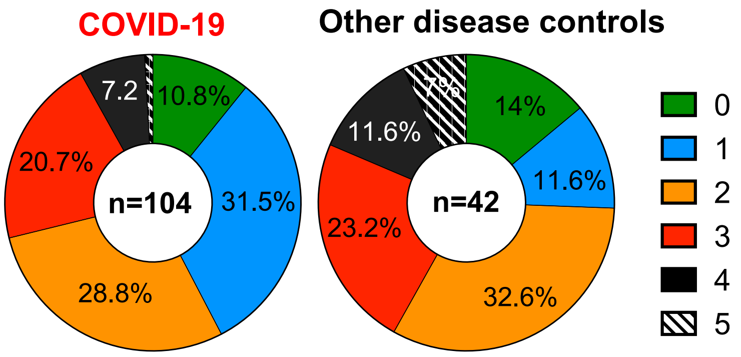

Extended Figure 1
Type, number, and frequency and types of co-morbidity present in COVID-19 and other disease control (ODC) participants.

Supplementary Figure 2

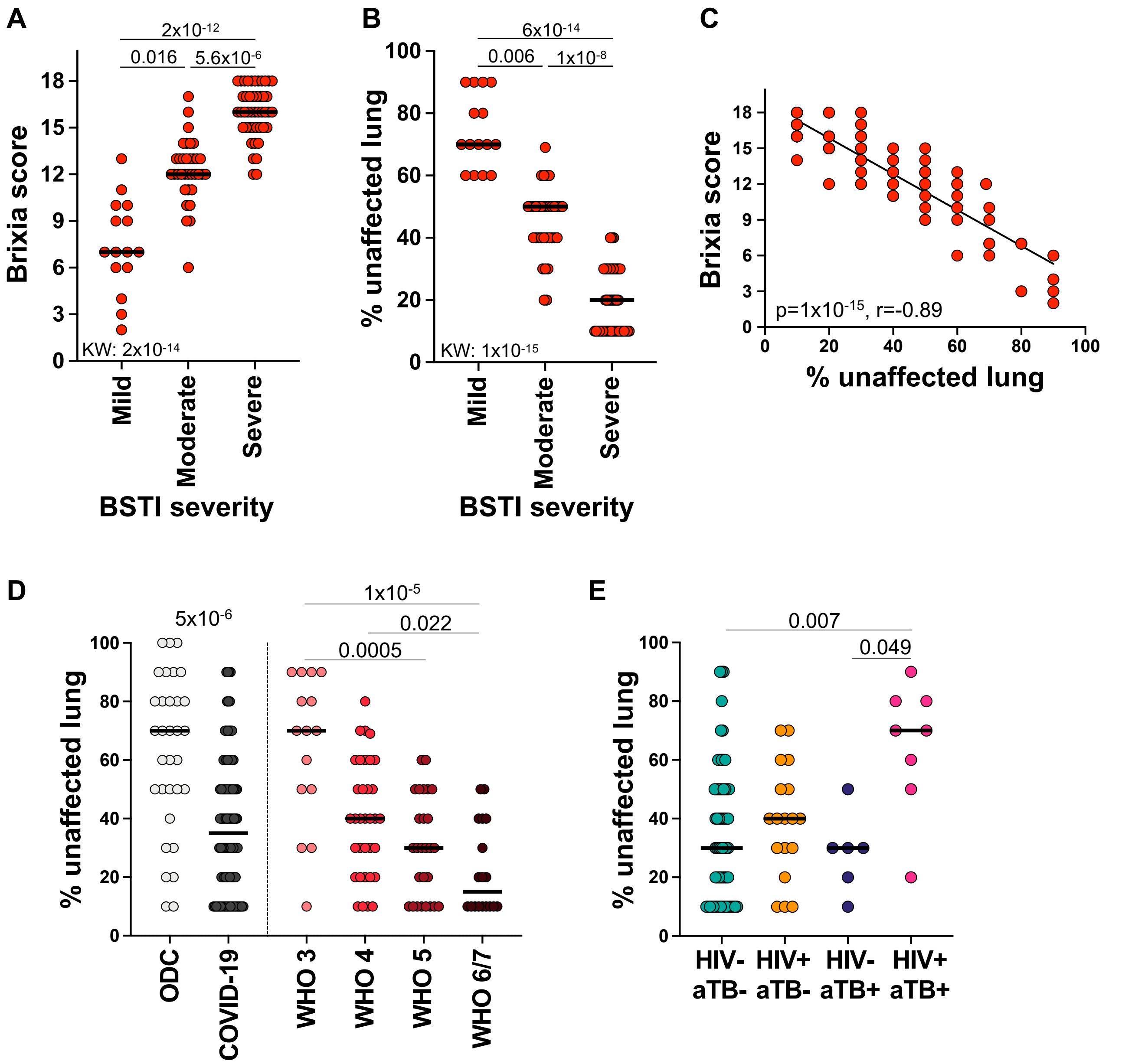

Extended Figure 2 Relationships between radiographic scores in n=104 SARS-CoV-2 patients

A Relationship between Brixia and British Society for Thoracic Imaging (BSTI) radiographic severity scores (3, 4).
B Inverse relationship between the BSTI radiographic severity score and the percentage unaffected lung.
C Inverse correlation between percentage of lung unaffected and Brixia severity score. Non-parametric Spearman correlation.
D Percentage unaffected lung scores in non-COVID-19 hospitalized patients (ODC) and COVID-19 patients and relationship between percentage unaffected lung and WHO clinical severity score
E Relationship between the absence (-) or presence (+) of HIV-1 and/or tuberculosis (TB) co-infection and percentage unaffected lung in COVID-19 patients.
Statistical comparisons were performed by the two-sided Kruskal-Wallis test (with Dunn's multiple comparison adjustments) in A, B, E and for the comparison of unaffected lung scores between patients grouped by WHO score. The comparison of OCD vs COVID-19 in D was done using a two-sided Mann-Whitney test.

Supplementary Figure 3

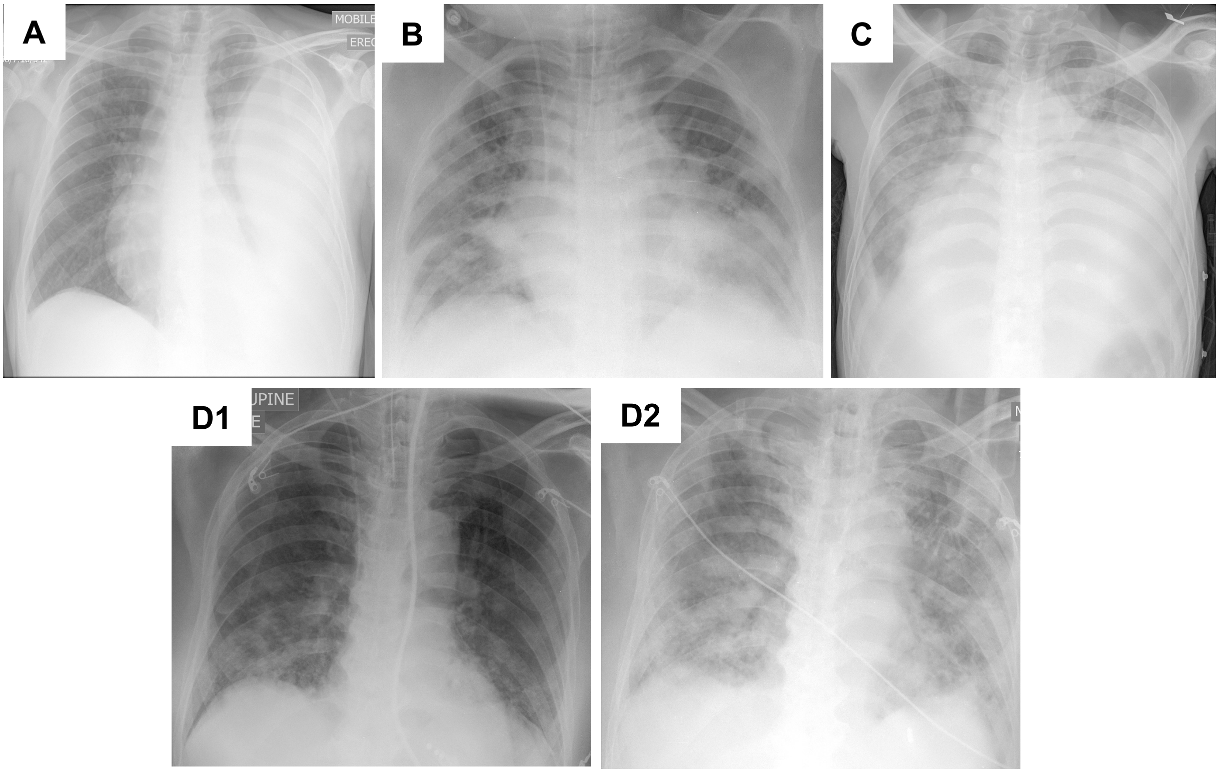

Extended Figure 3 Radiographic appearances of combined SARS-CoV2 and tuberculosis infection in the presence or absence of HIV-1 co-infection

A. 31-year-old HIV-1 infected antiretroviral-naive female (patient number 3) with a CD4 count of 106 cells/mm3 presenting with a large left pleural effusion that yielded a positive Gene Xpert MTB/Rif result. Also, SARS-CoV-2 RT-PCR positive with threshold cycle of 23.03. Uneventful course and discharged after 15 days on antitubercular therapy.

B. 41-year-old HIV-1 uninfected female patient number 73) with hypertension, obesity and type II diabetes mellitus. SARS-CoV-2 RT-PCR positive and WHO grade 6. Admitted to intensive care where a tracheal aspirate was Gene Xpert TB/Rif positive with rifampin resistance detected. The patient had a complicated course with *Candida albicans* and *Serratia marcescens* superinfections and died after 21 days.

C. 55-year-old HIV-1 infected antiretroviral-naive male (patient number 97)with a CD4 count of 17 cells/mm^3 presenting with a large pericardial effusion that was Gene Xpert MTB/Rif test positive. Also respiratory tract SARS-CoV-2 RT-PCR test was positive. Pericardiocentesis was performed and the patient was commenced on antitubercular therapy and discharged well to stepdown care after 7 days' admission.

D. 61-year-old HIV-1 uninfected male (patient number 80) with hypertension, obesity and type II diabetes mellitus presented ketoacidotic and in severe respiratory failure. SARS-CoV-2 RT-PCR positive. Intubated and ventilated. By day 25 of intensive care admission, radiographic deterioration prompted a Gene Xpert MTB/Rif test which was positive. The patient developed multiorgan failure and died on day 28 of admission

Supplementary Figure 4

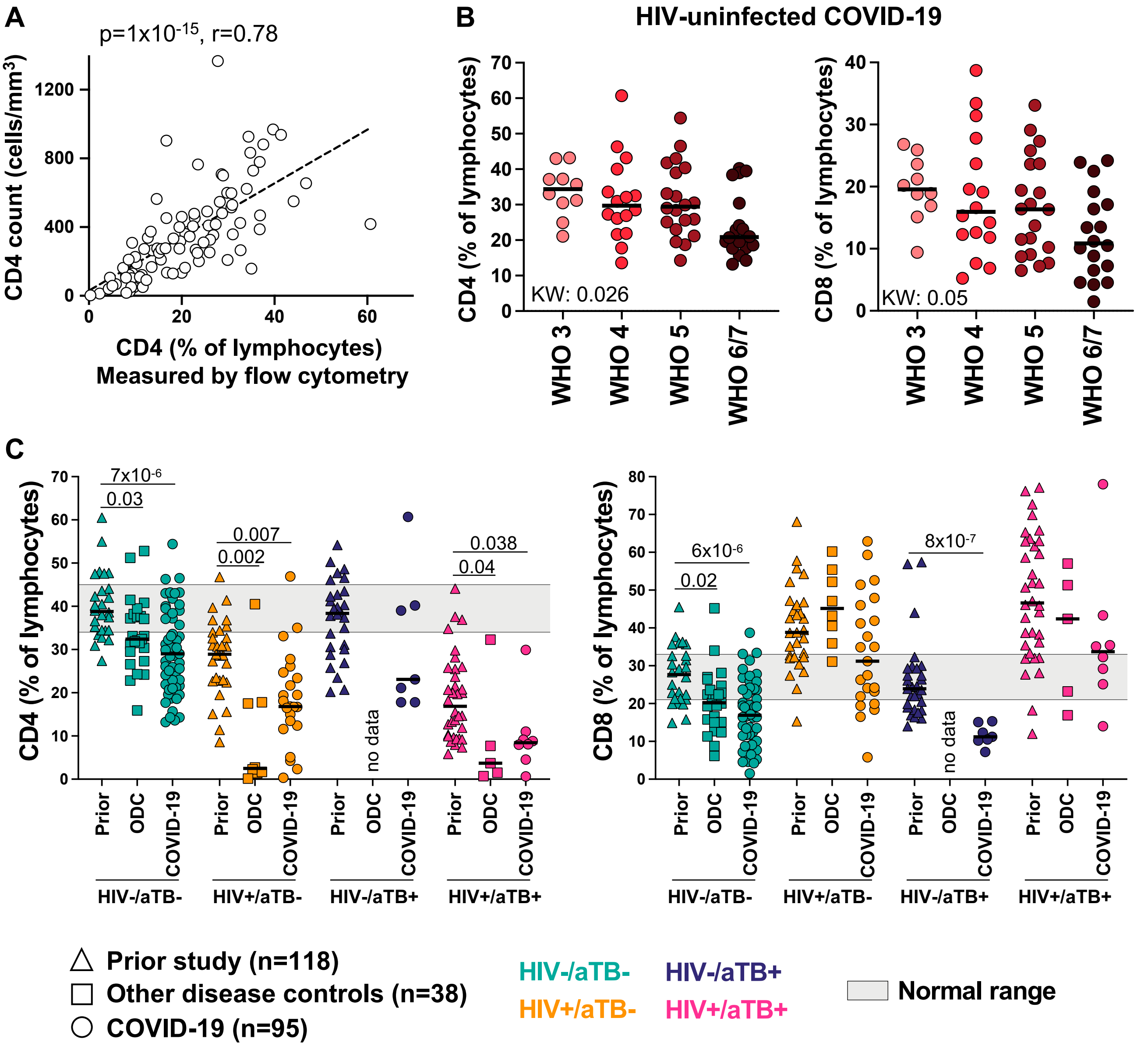

Extended Figure 4 Percentage of peripheral lymphocytes CD4 and CD8 positive in relation to COVID-19 severity and the presence or absence of HIV-1 and/or tuberculosis co-morbidities
A. Correlation between absolute CD4 lymphocyte count determined by coulter analysis and the CD4 percentage determined by flow cytometric analysis. Correlation was tested by a two-tailed non-parametric Spearman rank test.
B. Amongst n=73 HIV-1 uninfected patients with COVID-19 there was a trend towards a lower percentage of lymphocytes positive for CD4 (B) and CD8 T cells with increasing WHO grade severity, which was significant for CD4 (Kruskal Wallis test).
C. Additional control values were obtained from a subset of participants enrolled to a prior study of 118 ambulant HIV-1 uninfected and infected persons with either immune evidence of tuberculosis sensitization but no symptoms or microbiologically-confirmed pulmonary tuberculosis. When compared to HIV-1 uninfected healthy persons, the percentage of lymphocytes positive for CD4 was lower in both HIV-1 uninfected ODC and more so in COVID-19 patients. The pattern was reversed amongst HIV-1 infected patients with CD4 lymphocytes being especially low for ODC. Amongst patients with coincident HIV-1 and tuberculosis infection, % CD4 was very low irrespective of the presence or absence of SARS-CoV2 infection. When compared to HIV-1 uninfected healthy persons, the percentage of lymphocytes positive for CD8 was also lower in both HIV-1 uninfected ODC and more so in COVID-19 patients. However, this pattern was not observed amongst HIV-1 infected patients. The % CD8 positive lymphocyte was markedly depressed in HIV-1 uninfected patients with coincident tuberculosis and SARS-CoV2 infection. Comparisons between groups were performed using a Kruskal-Wallis test.

Supplementary Figure 5

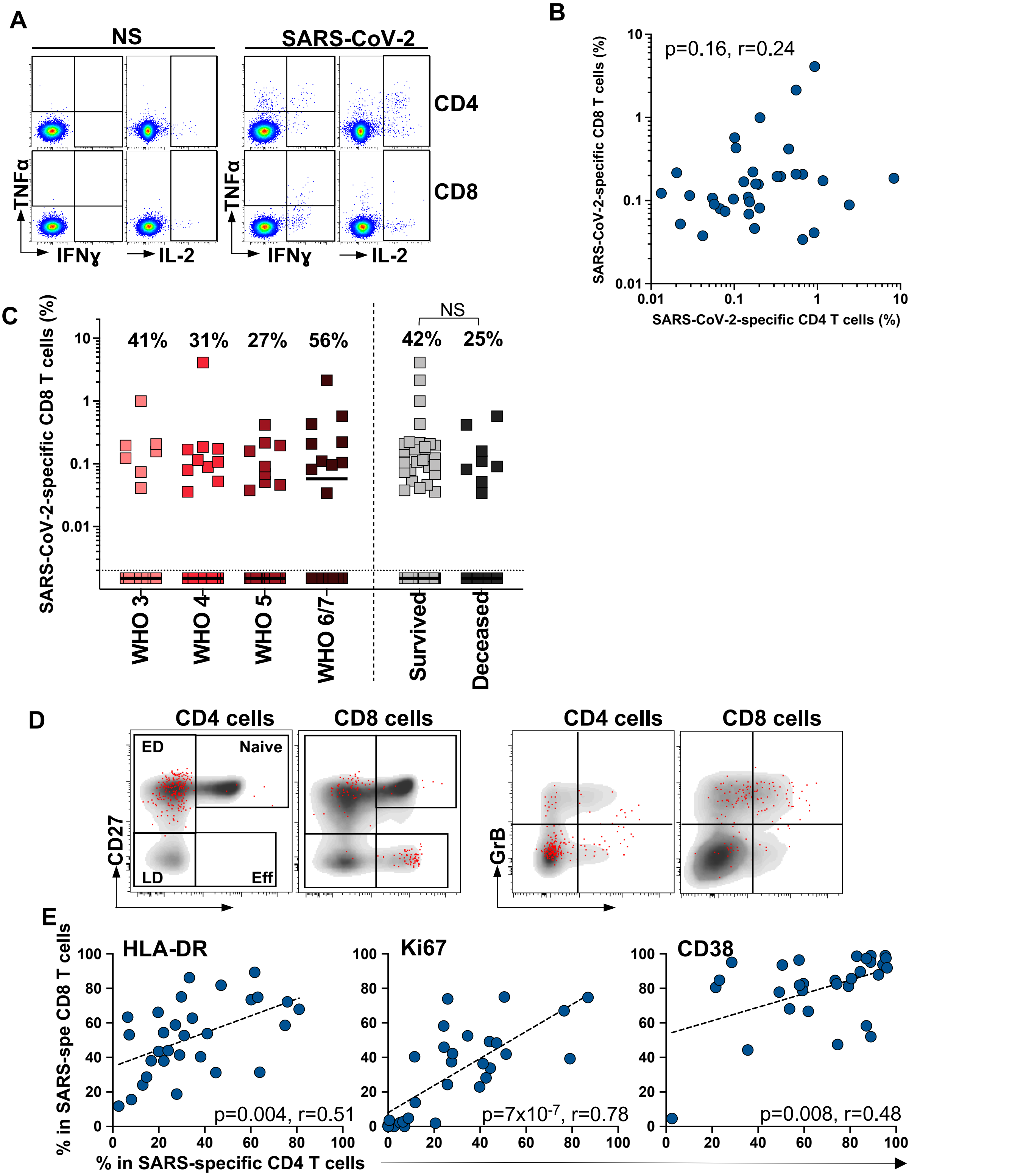

Extended Figure 5

A. Representative flow cytometry plots of IFN-ɣ, IL-2 and TNF-α expression in CD4 and CD8 T cells in responses to SARS-CoV-2 peptides. NS = no stimulation.

B. Association between SARS-CoV-2-specific CD4 and CD8 T cells in COVID-19 patients (n = 34). Correlation was tested by a two-tailed non-parametric Spearman rank test.

C. Prevalence and frequency of SARS-CoV-2-specific CD8 T cells in n=73 COVID-19 patients. Patients were stratified according to WHO ordinal score and outcome. Contingency analysis was performed by Chi-squared test

D. Overlay flow plots of CD45RA, CD27, Granzyme B, and HLA-DR expression. Red dots depict SARS-CoV-2-specific CD4 or CD8 T cells and density plots depict total CD4 or CD8 T cells. Four memory subsets can be delineated: Naive (CD45RA^{+}CD27^{+}), early differentiated (ED, CD45RA^{-}CD27^{+}), late differentiated (LD, CD45RA^{-}CD27^{-}) and effector (Eff, CD45RA^{+}CD27^{-})

E. Association between the expression of HLA-DR, Ki-67 and CD38 in SARS-CoV-2-specific CD8 T cells and SARS-CoV-2-specific CD4 T cells. Correlations were tested by two-tailed non-parametric Spearman rank test.
