# Supplementary material for: Effects of tuberculosis and/or HIV-1 infection on COVID-19 presentation and immune response in Africa
Source: Nat Commun. 2023 Jan 12;14:188. doi: 10.1038/s41467-022-35689-1 (PMC9836341; doi:10.1038/s41467-022-35689-1)
Supplement: Supplementary file 3 — Reporting Summary [file 41467_2022_35689_MOESM3_ESM.pdf]

nature portfolio

Corresponding author(s): Catherine Riou and Robert J Wilkinson

Last updated by author(s): Nov 14, 2022

Reporting Summary

Nature Portfolio wishes to improve the reproducibility of the work that we publish. This form provides structure for consistency and transparency in reporting. For further information on Nature Portfolio policies, see our Editorial Policies and the Editorial Policy Checklist.

Statistics

For all statistical analyses, confirm that the following items are present in the figure legend, table legend, main text, or Methods section.

n/a	Confirmed	
☐	☒	The exact sample size (*n*) for each experimental group/condition, given as a discrete number and unit of measurement
☒	☐	A statement on whether measurements were taken from distinct samples or whether the same sample was measured repeatedly
☐	☒	The statistical test(s) used AND whether they are one- or two-sided<br>*Only common tests should be described solely by name; describe more complex techniques in the Methods section.*
☐	☒	A description of all covariates tested
☐	☒	A description of any assumptions or corrections, such as tests of normality and adjustment for multiple comparisons
☐	☒	A full description of the statistical parameters including central tendency (e.g. means) or other basic estimates (e.g. regression coefficient) AND variation (e.g. standard deviation) or associated estimates of uncertainty (e.g. confidence intervals)
☐	☒	For null hypothesis testing, the test statistic (e.g. *F*, *t*, *r*) with confidence intervals, effect sizes, degrees of freedom and *P* value noted<br>*Give P values as exact values whenever suitable.*
☒	☐	For Bayesian analysis, information on the choice of priors and Markov chain Monte Carlo settings
☒	☐	For hierarchical and complex designs, identification of the appropriate level for tests and full reporting of outcomes
☐	☒	Estimates of effect sizes (e.g. Cohen's *d*, Pearson's *r*), indicating how they were calculated

Our web collection on statistics for biologists contains articles on many of the points above.

Software and code

Policy information about availability of computer code

Data collection	RedCap version XXX open source was used and this acknowledged in the methods
Data analysis	Flow cytometric analysis used FlowJo (v9.9.6, FlowJo LCC, Ashland, OR, USA). Other analyses used Analyses were conducted in Prism 8 (GraphPad Software, San Diego, CA)

For manuscripts utilizing custom algorithms or software that are central to the research but not yet described in published literature, software must be made available to editors and reviewers. We strongly encourage code deposition in a community repository (e.g. GitHub). See the Nature Portfolio guidelines for submitting code & software for further information.

Data

Policy information about availability of data

All manuscripts must include a data availability statement. This statement should provide the following information, where applicable:

- Accession codes, unique identifiers, or web links for publicly available datasets
- A description of any restrictions on data availability
- For clinical datasets or third party data, please ensure that the statement adheres to our policy

Raw data for figures and supplementary figures are provided as a supplementary .xls files.

Human research participants

Policy information about studies involving human research participants and Sex and Gender in Research.

Reporting on sex and gender	These data are provided in table 1. 70 males and 76 females were included. Assignment was on basis of participant self-identification
Population characteristics	These are described in table 1. This was a single-centre observational case-control study of adult (age > 18 years) patients admitted to Groote Schuur Hospital, Cape Town, South Africa with RT-PCR proven, or suspected, SARS-CoV-2 infection. Known HIV-1 status or willingness to undergo testing was an inclusion criterion. During the initial surge of hospital admissions recruitment could not be sequential due to caseload. Emphasis was on known SARS-CoV-2 PCR positive inpatients in a variety of dependency settings ranging through person under investigation (PUI), low dependency known positive, high-flow nasal oxygen and intensive care settings. As caseload decreased emphasis on PUI recruitment increased such that appropriate inpatient controls without laboratory evidence of SARS-CoV-2 infection (Non-COVID-19 participants, NC) could be recruited.
Recruitment	Between 11th June and 28th August 2020, the study team assessed suspected or confirmed SARS-CoV-2 (by nasopharyngeal or oropharyngeal aspirate) participants and expedited written electronic consent for inclusion was provided. Where participants did not have capacity to provide informed consent, e.g. in the case of those admitted in the intensive care unit, informed consent was sought from family members. Informed consent was provided by all participants who regained their capability to provide informed consent after incapacity. Known HIV-1 status or willingness to undergo testing was an inclusion criterion. During the initial surge of hospital admissions recruitment could not be sequential due to caseload. Emphasis was on known SARS-CoV-2 PCR positive inpatients in a variety of dependency settings ranging through person under investigation (PUI), low dependency known positive, high-flow nasal oxygen and intensive care settings. As caseload decreased emphasis on PUI recruitment increased such that appropriate inpatient controls without laboratory evidence of SARS-CoV-2 infection (Non-COVID-19 participants, NC) including seven who were not hospitalised could be recruited. The principal reason to include the NC was to evaluate the potential specificity of white cell counts and inflammatory markers in a contemporaneous group of patients. Self-selection bias is unlikely to have impacted results.
Ethics oversight	University of Cape Town Faculty of Health Sciences Research Ethical Committee

Note that full information on the approval of the study protocol must also be provided in the manuscript.

Field-specific reporting

Please select the one below that is the best fit for your research. If you are not sure, read the appropriate sections before making your selection.

☒ Life sciences ☐ Behavioural & social sciences ☐ Ecological, evolutionary & environmental sciences

For a reference copy of the document with all sections, see nature.com/documents/nr-reporting-summary-flat.pdf

Life sciences study design

All studies must disclose on these points even when the disclosure is negative.

Sample size	Sample size was exploratory
Data exclusions	Eight of the 50 RT-PCR negative NC participants tested SARS-CoV-2 antibody positive and were thus excluded from further analysis
Replication	Not applicable, this was a an observational study conducted at a unique timeduring the first wave of COVID-19 infection in Cape Town. Subsequent waves were caused by different variants of SARS-CoV-2
Randomization	Not applicable: no intervention was tested
Blinding	Not applicable: no intervention was tested

Reporting for specific materials, systems and methods

We require information from authors about some types of materials, experimental systems and methods used in many studies. Here, indicate whether each material, system or method listed is relevant to your study. If you are not sure if a list item applies to your research, read the appropriate section before selecting a response.

Materials & experimental systems

n/a	Involved in the study
☐	☒ Antibodies
☒	☐ Eukaryotic cell lines
☒	☐ Palaeontology and archaeology
☒	☐ Animals and other organisms
☒	☐ Clinical data
☒	☐ Dual use research of concern

Methods

n/a	Involved in the study
☒	☐ ChIP-seq
☐	☒ Flow cytometry
☒	☐ MRI-based neuroimaging

Antibodies

Antibodies used

- purified NA/LE mouse anti-human CD28 (clone 28.2), BD Pharmingen, Cat# 555725, RRID:AB_2130052, dilution: 1/1000
- purified NA/LE mouse anti-human CD49d (clone L25), BD Pharmingen, Cat# 555501, RRID:AB_396068, dilution: 1/1000
- CD3 BV650 (clone: OKT3), Biolegend, Cat# 317324, RRID:AB_11126748, dilution: 1/250
- CD4 BV785 (clone: OKT4), Biolegend, Cat# 317441, RRID:AB_2561365, dilution: 1/83
- CD8 BV510 (clone: RPA-8), Biolegend, Cat# 301048; RRID:AB_2561942, dilution 1/36
- CD45RA Alexa 488 (clone: HI100), Biolegend, Cat# 304114, RRID:AB_528816, dilution: 1/50
- CD27 PE-Cy5 (clone: 1A4CD27, Beckman Coulter), cat# 6607107, RRID:AB_10641617, dilution: 1/50
- CD38 APC (clone: HIT2) BD Biosciences, Cat# 555462, RRID:AB_398599, dilution: 1/8
- HLA-DR BV605 (clone L243), Biolegend, Cat# 307639, RRID:AB_11219187, dilution: 1/100
- Ki67 PerCP-Cy5.5 (clone B56), BD Biosciences, Cat# 561284, RRID:AB_10611574, dilution: 1/125
- PD-1 PE (Clone: J105) eBioscience), Cat# 12-2799-42, RRID:AB_11042478, dilution: 1/100
- Granzyme B (GrB) BV421 (clone: BG11, BD Biosciences), Cat# 563389, RRID:AB_2738175, dilution: 1/167
- IFN-ɣ BV711 (clone: 4S.B3), BioLegend Cat# 502540, RRID:AB_2563506, dilution: 1/71
- TNF-α PE-Cy7 (clone: MAB11) Biolegend, Cat# 502930, RRID:AB_2204079, dilution: 1/250

IL-2 PE/Dazzle 594 (clone: MQ1-17H12), Biolegend, Cat# 500344, RRID:AB_2564091, dilution: 1/71

Validation

All antibodies used in this study are commercially available. All antibodies were validated by their manufacturers and were titered to define the titer for optimal positive and negative separation

Flow Cytometry

Plots

Confirm that:

☒ The axis labels state the marker and fluorochrome used (e.g. CD4-FITC).

☒ The axis scales are clearly visible. Include numbers along axes only for bottom left plot of group (a 'group' is an analysis of identical markers).

☒ All plots are contour plots with outliers or pseudocolor plots.

☒ A numerical value for number of cells or percentage (with statistics) is provided.

Methodology

Sample preparation

Cell staining was performed on cryopreserved cells that were thawed, washed and permeabilized with a Transcription Factor perm/wash buffer (eBioscience). Cells were then stained at room temperature for 45 min with antibodies

Instrument

BD LSR-II

Software

FlowJo (v9.9.6, FlowJo LCC, Ashland, OR, USA). A positive response was defined as any cytokine response that was at least twice the background of unstimulated cells. To define the phenotype of SARS-CoV-2-specific CD4 T cells, a cut-off of 20 events was used.

Cell population abundance

A positive response was defined as any cytokine response that was at least twice the background of unstimulated cells. To define the phenotype of SARS-CoV-2-specific CD4 T cells, a cut-off of 20 events was used.

Gating strategy

Extended Figure 5A

☒ Tick this box to confirm that a figure exemplifying the gating strategy is provided in the Supplementary Information.

March 2021
